# Influence of Ultrasonic Impact Number on the Contact Fatigue Performance of Cr12Mo1V1 Die Steel Component

**DOI:** 10.3390/ma19143011

**Published:** 2026-07-13

**Authors:** Jian Wei, Qirong Xiao, Mengyu Cao, Hao Gao, Yuhong Liu, Chaoyu Li

**Affiliations:** 1 School of Mechanical and Electrical Engineering, Sanming University, Sanming 365004, China; 20240663114@fjsmu.edu.cn (J.W.);; 2 School of Mechanical Science and Engineering, Northeast Petroleum University, Daqing 163318, China; 3 Department of Engineering and Design, Sanming Medical and Polytechnic Vocational College, Sanming 365000, China

**Keywords:** Cr12Mo1V1 materials, ultrasonic impact number, die steel component, contact fatigue performance

## Abstract

In order to enhance the contact fatigue performance of the Cr12Mo1V1 die steel component, the ultrasonic impact surface-modification process was introduced into the heat treatment process of 1060 °C quenching + cryogenic treatment (−150 °C × 2 h) + two times of tempering at 520 °C × 2 h in this paper. The influence of the ultrasonic impact number on the surface morphology, roughness, micro-hardness value, residual stress, microscopic morphology of spalling pits, contact fatigue life, and EBSD inverse pole figure of the Cr12Mo1V1 die steel component were investigated utilizing a laser confocal microscope, 3D laser confocal microscope, micro-hardness tester, X-ray stress meter, scanning electron microscope, rolling contact fatigue testing machine, and EDAX-TSL system equipped with a field-emission scanning electron microscope, respectively. In comparison to other ultrasonic impact numbers, the Cr12Mo1V1 die steel component owned the smallest roughness of 0.027 μm, the highest micro-hardness value of 867 Hv_0.1_ and the largest residual compressive stress of −1318 MPa, respectively. Meanwhile, the contact fatigue life of the Cr12Mo1V1 die steel component was significantly improved by 223% to 5.231 × 10^7^ cycles. After ultrasonic impact treatment, the spalling pits revealed that the crack propagation angle increased from 28° to 34°, the depth increased from 153 μm to 175 μm, and the failure mode consisted of Hertz’s contact theory. This study provides an effective surface strengthening solution for enhancing the contact fatigue performance of Cr12Mo1V1 die steel components.

## 1. Introduction

Contact fatigue is a typical failure mode induced on the surfaces of workpieces under the long-term effect of alternating contact stress, primarily manifesting as pitting, spalling, and other forms of damage, leading to the service life of bearings, gears, and cold work molds decreased seriously [1,2,3]. Cr12Mo1V1 is one cold work die steel with high carbon–chromium and has excellent advantages of high micro-hardness (58–62 HRC) and wear resistance, and it is widely used in key components such as spinning wheels and stamping dies [4,5,6]. However, after quenching, this material contains a 10–30% amount of unstable retained austenite and is prone to strain-induced martensitic transformation under cyclic loading, resulting in local additional stress being formed and the contact fatigue resistance being significantly reduced [7,8,9]. Therefore, to reduce the content of retained austenite and improve the structural stability through phase transformation, strengthening is necessary. Meanwhile, the strain strengthening could be introduced to reduce surface roughness and inhibit the initiation and propagation of fatigue cracks to further improve the surface properties.

Cryogenic treatment is a thermal treatment process, which is usually cooled to temperatures below −70 °C (<203 K) to induce metallurgical changes to materials. Padmakumar et al. [10] reported the precipitation of eta-phase carbides, densification of a cobalt binder, refinement of tungsten carbide grains, phase transformation of cobalt, and changes in residual stress in cryogenically treated tungsten carbide (WC-Co) materials, which resulted in increased hardness and wear resistance. Kara et al. [11] studied the effects of cryogenic treatment on DIN 1.2344 hot work tool steel. In the study, the effects of shallow cryogenic treatment (SCT) and deep cryogenic treatment (DCT) at two different waiting times on the mechanical properties (microhardness, macrohardness, yield and tensile strength, elongation amount, impact energy) were investigated compared to the conventional heat-treated (CHT) sample. It was found that shallow and deep cryogenic treatment provided 1.34%, 9.31%, and 13% improvements in wear resistance for SCT-12, SCT-24, and DCT-36, respectively, compared to conventional heat treatment. In terms of impact resistance, the highest value was a 2.37% improvement in the DCT sample. Guili et al. [12] studied the effect of tempering temperatures on the microstructural composition and mechanical properties of AISI M35 high-speed steel subjected to deep cryogenic treatment. The carbide precipitation (secondary carbides and nanoscale carbides) began at a tempering temperature of 350 °C, increased at 450 °C, and reached its maximum at 550 °C. The impact toughness of the sample tempered at 550 °C was the best, namely 2.50 MJ m(−2), due to an increase in the number of martensite block boundaries and the more homogeneous carbide precipitation. Cracks occurred in the carbides with larger sizes (primary carbides and large secondary carbides) and at the carbide/matrix interface, and small secondary carbides decohered at the interface, forming micro-voids and facilitating plastic deformation.

Compared with deep cryogenic treatment, ultrasonic impact treatment can also improve the surface integrity and contact fatigue performance of materials. This technique induces severe plastic deformation in the surface layer of the material through high-frequency impacts of the tool head, achieving a “peak shaving and valley filling” surface finishing effect and significantly reducing surface roughness. Meanwhile, it generates a high-magnitude residual compressive stress field and a grain-refined strengthening layer in the surface layer, effectively inhibiting the initiation and propagation of fatigue cracks. Liu et al. [13] investigated the influence of building directions and ultrasonic surface rolling processing (USRP) on the rolling contact fatigue (RCF) performances of selective electron-beam-melted Ti6Al4V. The USRP-treated samples show similar to a 30% longer RCF life than that of untreated samples. The increased RCF performance is attributed to large work hardening, surface nano-crystallization, surface residual compressive stress, and the changed lubrication condition from boundary lubrication to mixed lubrication, which suppresses crack initiation and propagation. Hu et al. [14] investigated ultrasonic surface rolling (USR) on the material properties and rolling contact fatigue (RCF) behaviors of 25CrNi2MoV steel. USR treatment improved the surface quality (including roughness and oxide layer) of the DLSH samples, increased surface hardness, and obtained a beneficial surface compressive residual stress of up to 1240 ± 91 MPa. After USR treatment, the failure mode within the SZ changed from spalling to delamination, but it remained as spalling failure within the HZ. Luo et al. [15] analyzed the effect of temperature on the micro-structure and properties of the specimen by investigating the thermal stability of the Ti6Al4V alloy post-USRP (ultrasonic surface rolling process). The surface nano-hardness of the specimen after heat treatment at 450 °C reached a maximum value of 12.8 GPa, demonstrating optimal thermal stability. These findings indicate that enhancing surface layer properties through ultrasonic strengthening is another effective approach to improving the contact fatigue resistance of components.

The above analysis indicates that deep cryogenic treatment can stabilize the matrix micro-structure and increase hardness, while ultrasonic impact treatment can improve the surface stress state and surface quality. From a practical standpoint, the combination of deep cryogenic treatment (a bulk treatment) and ultrasonic impact treatment (a localized surface treatment) offers a cost-effective and industrially feasible route to enhance the service life of cold-work tool steels. The 0.2–0.3 mm surface modification layer with significantly improved hardness and compressive residual stress is particularly beneficial for components subjected to cyclic contact loading, where crack initiation typically occurs at the surface. As a result, the deep cryogenic treatment and ultrasonic impact treatment were synergistically combined in this paper. This study focuses on the strengthening mechanism and effects of Cr12Mo1V1 steel components subjected to quenching at 1060 °C + deep cryogenic treatment + two high-temperature tempers at 520 °C, which were selected as the test specimens, and optimization experiments for ultrasonic impact process parameters were carried out. The effects of reciprocating impact times of one, three, and six passes on the surface roughness, hardness gradient, residual stress distribution, and contact fatigue life of the components were investigated. The morphological characteristics and propagation behavior of fatigue spalling pits before and after composite modification were analyzed in depth. Finally, the synergistic strengthening mechanism of deep cryogenic treatment and ultrasonic impact was elucidated, providing a theoretical basis and process guidance for the performance improvement and life extension of Cr12Mo1V1 steel components under high-contact-stress conditions.

## 2. Materials and Methods

### 2.1. Preparation

**Figure 1:**
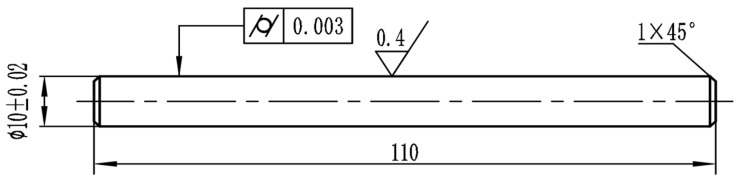
Specific size of Cr12Mo1V1 specimen used to test contact fatigue performance.

The industrial Cr12Mo1V1 was high-carbon, high-chromium, cold work die steel and selected as the experimental material. The main chemical composition of the Cr12Mo1V1 specimen is shown in Table 1. The specific size of the Cr12Mo1V1 specimen used to test contact fatigue performance is shown in Figure 1. Meanwhile, the surface roughness Ra was controlled below 0.8 μm to meet the requirements of subsequent fatigue tests.

**Figure 2:**
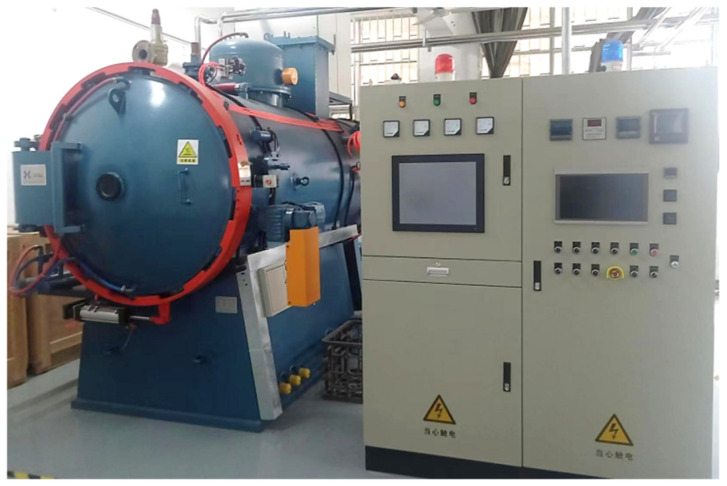
Experimental facility picture of double-chamber vacuum air-cooled oil quenching furnace.

**Figure 3:**
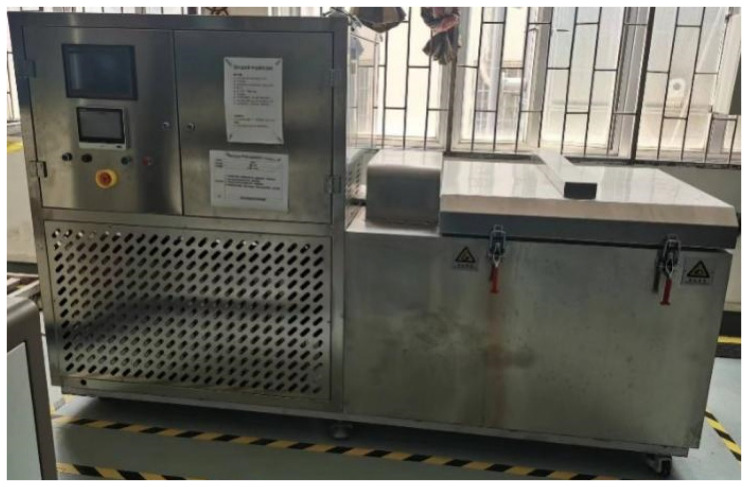
Experimental facility picture of liquid nitrogen hybrid deep freezer.

In this paper, the Cr12Mo1V1 specimen was treated using 1060 °C quenching + cryogenic treatment (−150 °C × 2 h) + tempering (520 °C × 2 h, twice). Figure 2 presents the experimental facility picture of the double-chamber vacuum air-cooled oil quenching furnace used to conduct quenching and tempering. The double-chamber vacuum air-cooled oil quenching furnace consisted of a furnace body, furnace door, heating system, vacuum system, cooling system, control system, and sealing system, respectively. Figure 3 reveals the experimental facility picture of the liquid nitrogen hybrid deep freezer used to conduct cryogenic treatment. The liquid nitrogen hybrid deep freezer mainly consisted of an inner box, outer box, electrical box, and upper cover, respectively. After the above treatment, the HKC30-50-type ultrasonic impact (Shandong Huayun Electromechanical Technology Co., Ltd., Jinan, China) equipment was employed for surface modification of the Cr12Mo1V1 specimen. All specimens were treated using an ultrasonic impact treatment (UIT) device with a tungsten carbide ball tip (3 mm diameter). The specific parameters of the ultrasonic impact process are listed as follows: vibration frequency 23 kHz, spindle speed 100 rpm, static load 700 N, traverse speed 0.1 mm/r, and unidirectional treatment direction (left to right). The specimens differ only in the number of cumulative passes:

USR-1 → 1 pass (total treatment time: 11 min)

USR-2 → 3 passes (total treatment time: 33 min)

USR-3 → 6 passes (total treatment time: 66 min)

Between consecutive passes, a 1 min dwell time was allowed to prevent thermal accumulation. The tool returned to the starting point after each pass and repeated the same unidirectional left-to-right path for all passes. No cross-directional passes were applied. Clamping torque and environmental conditions were kept constant for all specimens.

### 2.2. Characterization

The surface morphology and roughness of the Cr12Mo1V1 specimen were observed utilizing a laser confocal microscope (Zeiss, LSM900, Jena, Germany) and a 3D laser confocal microscope (Keyence, VK-X1000K, Osaka, Japan), respectively. The micro-hardness value of the Cr12Mo1V1 specimen was investigated utilizing a micro-hardness tester (FALCON-500, The Dutch company INNOVATEST, Maastricht, The Netherlands) with the operation condition of a 100 g load and kept for 10 s. Meanwhile, six points were measured from 0.1 mm, 0.2 mm, 0.5 mm, 1.0 mm, 1.5 mm, and 2.0 mm along the specimen surface, and a Gradient test was conducted along the depth direction. In order to enhance accuracy, each position was measured three times and averaged.

According to the relevant standard ASTM E975-03 [16] and E2860-12 standards [17], the retained austenite content and the residual stress of the Cr12Mo1V1 specimen were measured using an HDS-I X-ray stress meter (Haidexing Technology Co., Ltd., Xiamen, China). Also, the lateral tilt fixation (Ψ) method and electrolytic layer removal method was employed to obtain the distribution characteristics of residual stress along the depth. Unlike mechanical polishing, it removes material layer-by-layer via anodic dissolution in an electrolyte. For deep-profile analysis (penetrating from the surface down to several hundred micrometers or even millimeters), this method is preferred because it does not introduce mechanical work hardening or compressive stress artifacts that would alter the underlying stress state. The EBSD analysis was conducted on an EDAX-TSL system (TexSEM Laboratories, Inc., Provo, UT, USA) equipped with a field-emission scanning electron microscope (FE-SEM).

In order to improve the reliability of the results, three parallel specimens were used for each test condition. The roughness, hardness, residual stress, and fatigue life were measured on all three specimens, and the average values of these measurements were reported as the final results.

**Figure 4:**
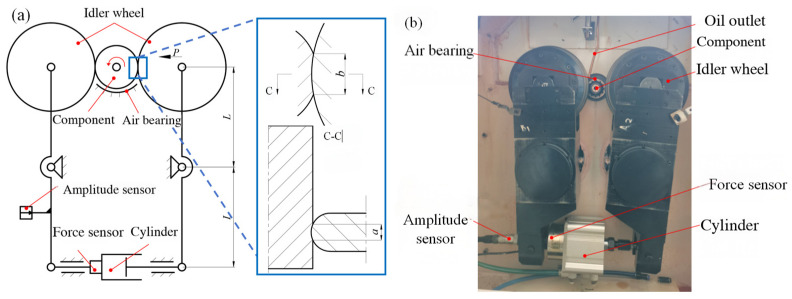
(**a**) Schematic diagram and (**b**) experimental equipment picture of contact fatigue test device. The arrow indicates the direction of rotation of the component.

Figure 4 displays the contact fatigue performance of the Cr12Mo1V1 specimen, which was assessed using an RCTF-6000 rolling contact fatigue testing machine (Beijing Aviation Precision Machinery Research Institute, Beijing, China). The component is clamped on the air spindle between two pressing idlers. The interaction between the two idlers ensures that the component does not bear additional bending loads, and the component drives the two pressing idlers to perform pure rolling. The cylinder applies a contact load p to the component through the swing arm, as shown in Figure 4. The applied load p equals the thrust of the cylinder, and its applied load is detected by a pressure sensor mounted on the cylinder. An amplitude sensor is mounted on the swing arm. When the component exhibits contact fatigue damage and spalling pits, the system will exhibit certain vibrations. The test will automatically stop when the amplitude reaches a set threshold. The contact stress (P_0_) of the Cr12Mo1V1 specimen was calculated using Equation (1):

(1) $$P_{0}=\frac{3}{2}\frac{P}{\pi ab}$$

where a was the half-length, b was the semi-width contact, and P was the total load, respectively.

To accelerate the testing process, a contact stress of 4 GPa and a spindle speed of 12,000 r/min are selected here. The contact stress of 4 GPa was selected based on both the typical service conditions of cold-work die steels (2–5 GPa in practical applications) and preliminary trial tests, which indicated that this stress level provides a discriminative baseline fatigue life (approximately 3.5 × 10^5^ cycles for untreated specimens) while allowing effective comparisons among different treatment conditions. The spindle speed of 12,000 rpm (200 Hz) was chosen following the recommendations of ISO 1143 [18] and ASTM E466 [19], as well as common practice in the literature, to ensure testing efficiency and result comparability. When the component exhibits spalling pits and the amplitude sensor detects an amplitude of 10 μm in the swing arm, the machine will automatically stop. Subsequently, a Zeiss Crossbeam 550 scanning electron microscope and a 3D laser confocal microscope are used to observe the morphology of the spalling pits and measure characteristic parameters such as the depth, angle, and area of the spalling pits.

## 3. Results and Discussion

### 3.1. Surface Morphology and Roughness Value Investigation

**Figure 5:**
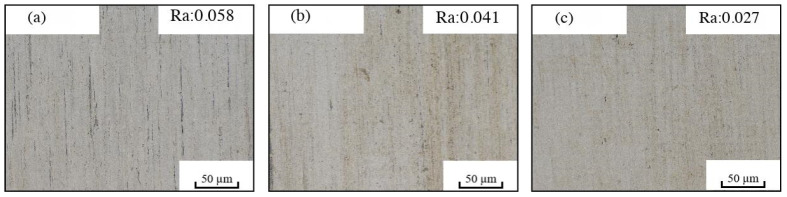
Surface morphology of Cr12Mo1V1 die steel components after ultrasonic impact numbers: (**a**) USR-1 specimen, (**b**) USR-2 specimen, and (**c**) USR-3 specimen.

Figure 5 presents the surface morphology of Cr12Mo1V1 die steel components after ultrasonic impact numbers. The obvious processing grooves existed on the surface of the USR-1 specimen, while the slight processing grooves existed on the surface of the USR-2 specimen. By comparison, the processing grooves of the USR-3 specimen almost completely disappeared and presented a uniform and smooth morphology. The results contributed to increasing the number of ultrasonic impacts, which could continuously enhance the smoothing effect of “peak shaving and valley filling”. The conclusion was similar to the findings of Li et al. [20] and Cui et al. [21].

**Figure 6:**
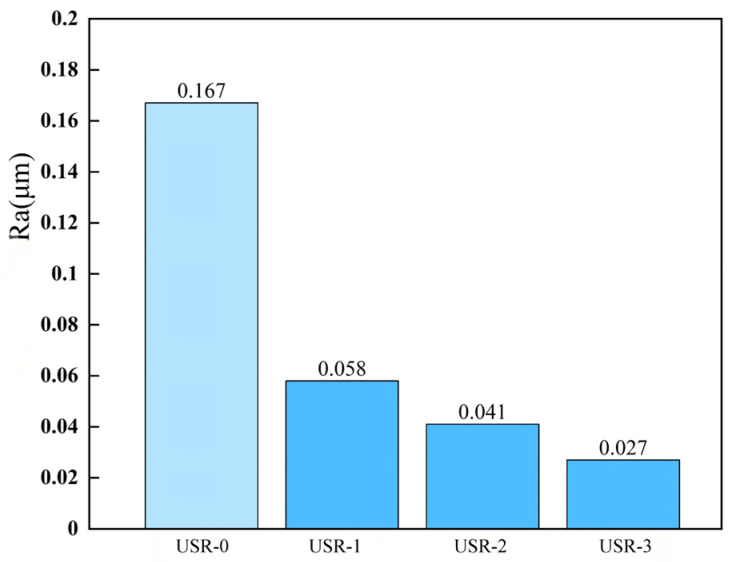
Roughness value of Cr12Mo1V1 die steel components after ultrasonic impact numbers.

Figure 6 exhibits the roughness values of Cr12Mo1V1 die steel components after ultrasonic impact numbers. The roughness values of USR-0, USR-1, USR-2, and USR-3 specimens were 0.167 μm, 0.058 μm, 0.041 μm, and 0.027 μm, respectively. The significant variation in roughness values is primarily attributed to the action of ultrasonic impact, which could flatten the surface wave peaks and fill the wave troughs, thereby achieving surface smoothing [22]. As the number of ultrasonic impacts increased, the plastic deformation layer gradually accumulated and the degree of work hardening intensified. Each impact further flattens the residual protrusions based on the previous one, making the surface tend towards absolute flatness. Therefore, the surface roughness of Cr12Mo1V1 die steel components was continuously reduced. The deep cryogenic treatment of components provides an ideal organizational foundation for this process. This technique promoted the uniform precipitation of carbides and reduced lattice distortion in the martensitic matrix, forming a uniform structure with good plasticity and stress relaxation. Under the positive synergy of “uniform structure” and “multiple impacts”, the former ensures the uniformity of deformation, while the latter provides sufficient cumulative energy, jointly contributing to the effect of reducing roughness.

### 3.2. Microstructure

**Figure 7:**
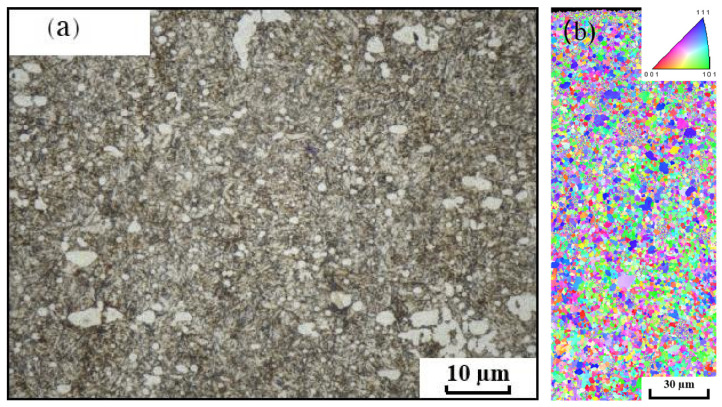
Microstructure of Cr12Mo1V1 die steel components after six ultrasonic impacts: (**a**) USR-3 equivalent component metallographic structure, (**b**) USR-3 equivalent component EBSD inverse pole figure.

Following the experimental procedures detailed in Section 2, cross-sectional specimens of the equivalent components subjected to various composite modification processes were prepared and examined via confocal microscopy. Figure 7a displays the representative metallographic structure of the USR-3 equivalent component, in which a typical microstructure consisting of acicular martensite, retained austenite, and dispersed carbides is clearly resolved. The presence of these constituent phases is consistent with the expected microstructural features of the Cr12Mo1V1 steel after deep cryogenic treatment, while the morphological characteristics of each phase provide initial indications of the microstructural modifications induced by the combined treatment.

To gain further insight into the underlying mechanisms governing the microstructural evolution, more detailed characterization was carried out on the USR-3 specimens. The primary objective was to elucidate how the synergistic application of deep cryogenic treatment and ultrasonic impact modifies the metallographic structure, particularly with respect to phase transformation, grain refinement, and carbide behavior. The cryogenic treatment is expected to promote the transformation of metastable retained austenite into martensite, thereby increasing the martensite fraction and contributing to transformation strengthening. Concurrently, the ultrasonic impact introduces severe plastic deformation into the near-surface region, leading to dislocation accumulation and grain subdivision, which are manifested as morphological changes in the surface layer. The combined effect of these two mechanisms is believed to be responsible for the enhanced mechanical performance observed in the USR-3 specimens, as will be further substantiated by the subsequent microstructural and mechanical property analyses.

Figure 7b displays the EBSD characterization of the USR-3 equivalent component, which provides compelling microstructural evidence of the synergistic strengthening mechanism induced by the combined cryogenic and ultrasonic impact treatment. In the IPF map, grains in red color have their <001> axis being close to the extrusion direction, while grains in blue or green colors have their <001> axis being nearly perpendicular to the extrusion direction. Large lattice distortion is observed, suggesting a high density of dislocations.

Of particular interest is the observation that the combined treatment yields a ~30% increase in the effective deformation layer thickness (reaching ~200 µm) compared with the ultrasonic impact treatment alone. This enhancement is attributed to the prior cryogenic treatment, which substantially reduces the retained austenite content from 21.3% to 2.6% and concomitantly increases the matrix strength. A stronger matrix is believed to be more resistant to localized softening and crack initiation during high-frequency impact, thereby allowing the more efficient transfer and distribution of plastic strain into deeper subsurface layers. Simultaneously, the increased matrix strength promotes a higher rate of dislocation accumulation, as evidenced by the higher hardness values in the surface region of the combined-treated specimens.

Taken together, the microstructural observations suggest that two distinct yet complementary strengthening mechanisms are operative in the combined-treated material: (i) transformation strengthening, accomplished by deep cryogenic treatment through the near-complete elimination of metastable retained austenite, which provides a hardened and volumetrically stable matrix; and (ii) dislocation strengthening, introduced by the ultrasonic impact, which creates a high-density dislocation network and nanograined surface layer. The interplay between these two mechanisms—where the cryogenically hardened matrix facilitates more effective dislocation storage and deformation localization—accounts for the superior surface mechanical properties and the extended fatigue life observed in the combined-treated specimens. This synergistic effect highlights the advantage of coupling a bulk-phase transformation treatment with a local surface severe plastic deformation technique for optimizing the performance of the Cr12Mo1V1 tool steel.

### 3.3. Micro-Hardness Value Detection

**Figure 8:**
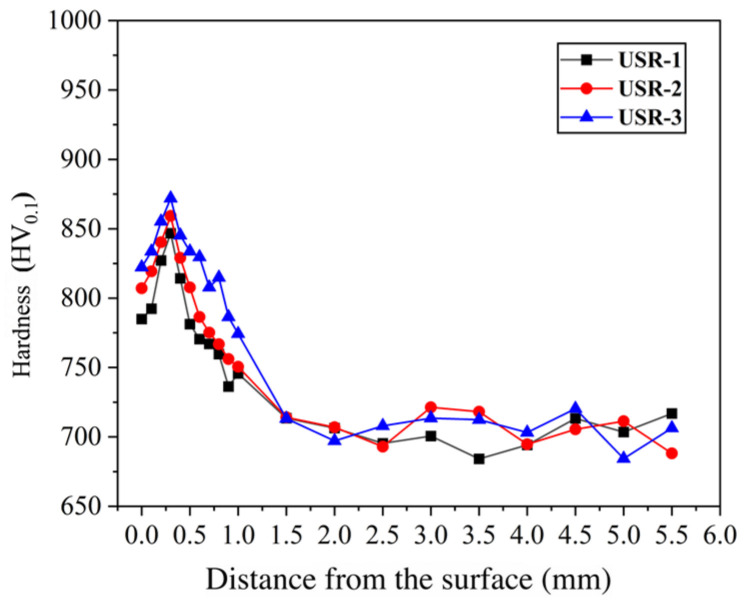
Micro-hardness value of Cr12Mo1V1 die steel components after ultrasonic impact numbers.

The micro-hardness values of Cr12Mo1V1 die steel components after ultrasonic impact numbers are displayed in Figure 8. After ultrasonic impact, the surface micro-hardness value of the Cr12Mo1V1 die steel component significantly increased compared to that of the untreated sample. Meanwhile, the maximum micro-hardness value of the Cr12Mo1V1 specimen was obtained at a depth of 0.3 mm from the surface, and then, it gradually decreased with increasing depth until it matched the matrix micro-hardness value. The maximum micro-hardness values of USR-1, USR-2, and USR-3 specimens are approximately 846 HV0.1, 859 HV0.1, and 872 HV0.1, respectively. Compared with the untreated sample, all treated specimens exhibited varying degrees of hardness enhancement. Specifically, the average hardness values of USR-1, USR-2, and USR-3 within a 0.3 mm depth from the surface increased by 19.4%, 21.2%, and 23.1% that of USR-0 (708.52 HV_0.1_).

The reason for this result could be explained as follows: (1) the deep cryogenic treatment promotes the transformation of residual austenite into martensite through phase transformation strengthening, increasing lattice distortion, and thereby significantly enhancing the micro-hardness value of the material [23]. (2) During the ultrasonic impact process, the tool head applies high-frequency impact loads to the surface of the component, causing plastic deformation of the surface material and generating a large number of dislocations. The plastic strain intensifies lattice distortion, further increasing the micro-hardness value of the surface layer [24]. The dislocations intertwined and accumulated with each other and formed a high-density dislocation structure, which hindered the subsequent plastic deformation and increased the micro-hardness value [25]. The increasing number of ultrasonic impacts intensified the degree of plastic deformation, leading to the dislocation density further increasing and generating the high micro-hardness value. (3) In addition, ultrasonic impact may also cause the surface grains to become finer and further increase the micro-hardness value [26].

### 3.4. Residual Stress Analysis

**Figure 9:**
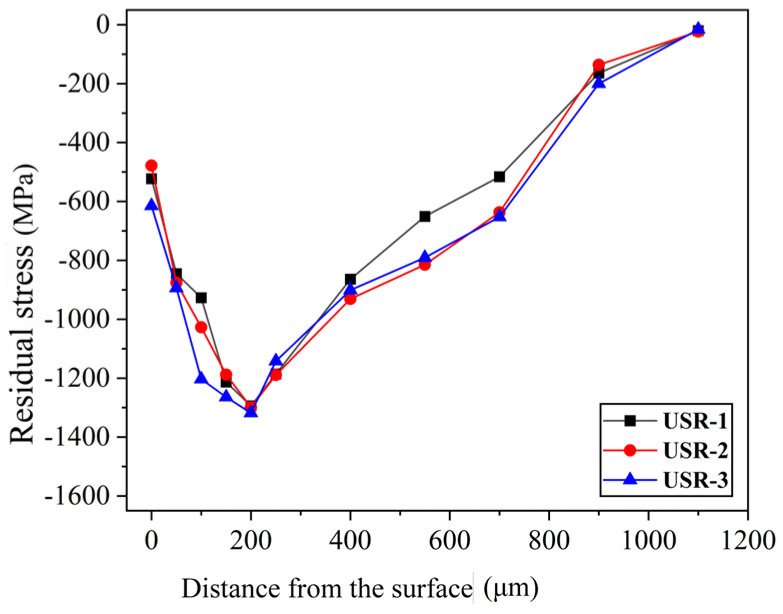
Residual stress of Cr12Mo1V1 die steel components after ultrasonic impact numbers.

The residual stress of Cr12Mo1V1 die steel components after ultrasonic impact numbers are revealed in Figure 9. The maximum residual stress of Cr12Mo1V1 specimens increased with the number of ultrasonic impacts. The maximum residual stress of USR-1, USR-2, and USR-3 specimens were −1294, −1299, and −1318 MPa, respectively. Compared with the untreated sample, all treated specimens exhibited varying degrees of residual stress enhancement. Specifically, the average residual stress values of USR-1, USR-2, and USR-3 within a 0.2 mm depth from the surface increased by 8.51, 8.55, and 8.69 times that of USR-0 (−136 MPa), respectively. According to Hertz contact theory [27], the maximum residual stress occurs at the position with the greatest contact deformation, which is consistent with the experimental results.

The variation in the maximum residual stress of Cr12Mo1V1 specimens was significantly influenced by the number of ultrasonic impacts. During the ultrasonic impact process, the tool head will cause plastic deformation on the surface of Cr12Mo1V1 specimens and prevent the material surface from rebounding after the impact, leading to the residual stress on the surface of Cr12Mo1V1 specimens. Therefore, the multiple number of ultrasonic impact resulted in the gradual accumulation of plastic deformation and was accompanied by the cold work hardening effect [28]. Meanwhile, the residual stress was difficult to be released through natural aging, leading to the accumulated residual stress of Cr12Mo1V1 specimens being gradually increased [29].

### 3.5. Contact Fatigue Performance Analysis

All fatigue tests of Cr12Mo1V1 die steel components after ultrasonic impact numbers were conducted on three independent specimens for each treatment condition, and the results are reported as mean values, which are displayed in Table 2. The experimental data indicated that the mean contact fatigue lives of USR-1, USR-2, andUSR-3 specimens were obviously higher than that of the USR-0 specimen. The contact fatigue lives of USR-1, USR-2, and USR-3 specimens were 3.765 × 10^7^, 4.767 × 10^7^, and 5.231 × 10^7^ cycles, respectively, corresponding to 2.33, 2.95, and 3.23 times that of USR-0 (1.618 × 10^7^ cycles).

The variation reason of contact fatigue life could be attributed to the synergistic effect of roughness, micro-hardness value, and residual stress. Firstly, the large number of ultrasonic impacts could significantly reduce roughness of the Cr12Mo1V1 specimen. Then, the large number of ultrasonic impacts contributed to the formation of the high micro-hardness value and high residual stress. Finally, the synergistic effect of these factors effectively suppressed the initiation and propagation of fatigue cracks, thereby significantly enhancing the contact fatigue life of the Cr12Mo1V1 specimen.

In summary, the ultrasonic impact treatment significantly improves the contact fatigue life through the synergistic effects of compressive residual stress, work hardening, and surface roughness reduction.

Compressive residual stress acts as the primary contributor by reducing the effective tensile stress at the subsurface and retarding crack initiation. Work hardening (microhardness increase) enhances the resistance to plastic deformation and suppresses the accumulation of fatigue damage. The surface roughness reduction plays a secondary but non-negligible role by eliminating stress concentration sites that could promote premature crack nucleation.

We have also included a quantitative comparison showing that the specimen with the highest compressive residual stress and hardness (USR-3) exhibits the longest fatigue life, while the untreated specimen (USR-0) shows the poorest performance. This correlation confirms that the fatigue life improvement is governed by the synergistic interaction of all three factors, with compressive residual stress being the dominant mechanism.

### 3.6. Microscopic Morphology of Spalling Pits Observation

**Figure 10:**
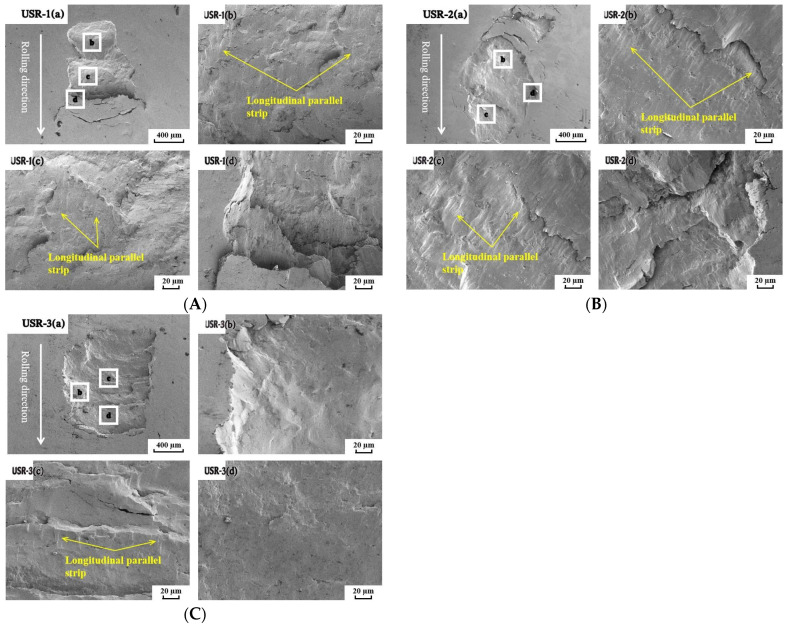
Spalling pit morphology of (**A**) USR-1 specimen, (**B**) USR-2 specimen, and (**C**) USR-3 specimen.

The spalling pit morphology of Cr12Mo1V1 die steel components after ultrasonic impact numbers is shown in Figure 10. The high-magnified SEM image of the USR-1 specimen presented the obvious ring-shaped band features, and the original processing features were not completely eliminated. Meanwhile, the small tearing ridges and tiny pores appeared on the spalling pit edge of the USR-2 specimen, which confirmed the characteristics of ductile fracture. The spalling pit edge of the USR-3 specimen still showed ductile fracture, and the original processing marks were completely covered.

Furthermore, the bottom morphology of the spalling pits of the USR-1 specimen was relatively smooth, with visible longitudinal parallel strips and sharp edges, which was obvious brittle-fracture characteristics. The bottom morphology of the spalling pits of the USR-2 specimen was more smooth and flat, with more obvious longitudinal parallel strips, demonstrating that the surface quality was significantly improved. The bottom morphology of the spalling pits of the USR-3 specimen presented a nearly smooth state and achieved a “peak reduction and valley filling” effect. Meanwhile, the deep residual stress field (reaching to hundreds of micrometers) and a gradient nano-crystalline layer are introduced, which could suppress crack initiation and generate shallow and small spalling pits [30]. In summary, the synergistic effect of surface-integrity enhancement and microstructure reconstruction of the subsurface significantly improved the fatigue resistance of materials [31]. Furthermore, the gradient nanocrystalline layer generated a high micro-hardness value and strength, which inhibit dislocation movement and delay crack propagation [32]. Also, the depth matching formed between the residual compressive stress field and the nano-crystalline layer constituted a multi-scale damage-resistant barrier [33].

**Figure 11:**
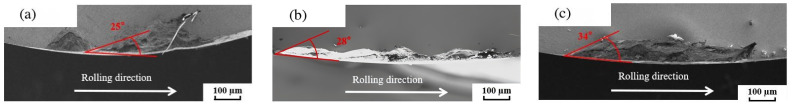
Cross-sectional morphology of spalling pits from Cr12Mo1V1 die steel components after ultrasonic impact numbers. (**a**) USR-1 specimen, (**b**) USR-2 specimen, and (**c**) USR-3 specimen.

The cross-sectional morphology of spalling pits from Cr12Mo1V1 die steel components after ultrasonic impact numbers are shown in Figure 11. It could be clearly seen that the expansion angles of cracks were significantly influenced by the number of ultrasonic impacts. As the number of ultrasonic impacts increased from one to six, the angle of spalling pits rose from 24° to 31°. The angle variation of spalling pits could be explained by the ultrasonic impact causing the concentration areas of surface stress to expand and connect, resulting in cracks being inclined to extend along the path with the least residual stress during the contact fatigue process [34]. The findings indicated that the increasing the number of ultrasonic impacts could effectively optimize the propagation path of cracks and change the fracture behavior of the material.

## 4. Conclusions

(1) The ultrasonic impact number could significantly improve the surface properties of Cr12Mo1V1 die steel components. Experimental results shown that the large ultrasonic impact number could significantly reduce the surface roughness and increase the micro-hardness value of components. Meanwhile, the large ultrasonic impact number established a high-amplitude residual stress field, which affected a depth of up to hundreds of micrometers. In addition, the high ultrasonic impact number could achieve “peak shaving and valley filling” on the surface of the component, significantly enhancing the surface integrity and mechanical properties.
(2) The contact fatigue life of Cr12Mo1V1 die steel components was significantly enhanced via a composite modification technique. In comparison to other components, the one treated after an ultrasonic impact number of six had the highest contact fatigue life of 5.231 × 10^7^ times, which was attributed to the synergistic effects of reduced surface roughness, the establishment of a residual compressive stress field, and work hardening.
(3) The failure mode and fracture behavior of Cr12Mo1V1 die steel components were changed by the ultrasonic impact number. The morphology analysis of spalling pits revealed that the increasing number of ultrasonic impacts resulted in expansion angle increases from 28° to 31°, which is closer to the maximum shear direction predicted by Hertz’s contact theory. Moreover, SEM images indicated that the ultrasonic impact number decreased the striped marks of the spalling pit bottom and weakened layered characteristics and the sparse crack distribution, indicating that the fracture mechanism of the material was optimized.

## Figures and Tables

**Table 1:** The elemental composition of Cr12Mo1V1 specimen.

Element	C	Si	Mn	P	S	Cr	Mo	V	Co	Fe
Content (wt%)	1.4	0.6	0.6	0.03	0.03	12.0	1.0	1.0	1.0	Balance

**Table 2:** Contact fatigue life of Cr12Mo1V1 die steel components after ultrasonic impact numbers.

Specimens	Contact Fatigue Life (×10^7^)
USR-0	1.618
USR-1	3.765
USR-2	4.767
USR-3	5.231

## Data Availability

The original contributions presented in this study are included in the article. Further inquiries can be directed to the corresponding authors.
